# Microbial Communities Associated With Passive Acidic Abandoned Coal Mine Remediation

**DOI:** 10.3389/fmicb.2019.01955

**Published:** 2019-08-23

**Authors:** Truc Ly, Justin R. Wright, Nicholas Weit, Christopher J. McLimans, Nikea Ulrich, Vasily Tokarev, Michelle M. Valkanas, Nancy Trun, Shawn Rummel, Christopher J. Grant, Regina Lamendella

**Affiliations:** ^1^Department of Biology, Juniata College, Huntingdon, PA, United States; ^2^Wright Labs LLC., Huntingdon, PA, United States; ^3^Department of Biological Sciences, Duquesne University, Pittsburgh, PA, United States; ^4^Trout Unlimited, Arlington, VA, United States

**Keywords:** acid mine drainage, sulfate reducing bacteria, passive remediation system, shotgun-metagenomics, 16S rRNA

## Abstract

Acid mine drainage (AMD) is an environmental issue that can be characterized by either acidic or circumneutral pH and high dissolved metal content in contaminated waters. It is estimated to affect roughly 3000 miles of waterways within the state of Pennsylvania, with half being acidic and half being circumneutral. To negate the harmful effects of AMD, ∼300 passive remediation systems have been constructed within the state of Pennsylvania. In this study, we evaluated the microbial community structure and functional capability associated with Middle Branch passive remediation system in central PA. Sediment and water samples were collected from each area within the passive remediation system and its receiving stream. Environmental parameters associated with the remediation system were found to explain a significant amount of variation in microbial community structure. This study revealed shifts in microbial community structure from acidophilic bacteria in raw AMD discharge to a more metabolically diverse set of taxa (i.e., Acidimicrobiales, Rhizobiales, *Chthoniobacteraceae*) toward the end of the system. Vertical flow ponds and the aerobic wetland showed strong metabolic capability for sulfur redox environments. These findings are integral to the understanding of designing effective passive remediation systems because it provides insight as to how certain bacteria [sulfate reducing bacteria (SRBs) and sulfur oxidizing bacteria (SOBs)] are potentially contributing to a microbially mediated AMD remediation process. This study further supports previous investigations that demonstrated the effectiveness of SRBs in the process of removing sulfate and heavy metals from contaminated water.

## Introduction

Acid mine drainage (AMD) is both an economic and ecological issue which results from the oxidized mine tailings of mining operations prior to the Surface Mining Control and Reclamation Act of 1977. Economic issues include a decline in property value and corroded man-made structures (i.e., well casings) and aquifers from exposure to AMD, resulting in harmful sources of water and recreational activities. Additionally, remediation costs for Pennsylvania have been estimated to be several billion United States dollars (Feasby et al., 1991). AMD is generated through a series of oxidation events of sulfide minerals, primarily iron pyrite, yielding sulfuric acid and hydrogen ions (Akcil and Koldas, 2006). AMD can be acidic or circumneutral and contain high levels of heavy metals and sulfate (Ferris et al., 1989). Acidic AMD frequently contains high concentrations of aluminum and manganese, while circumneutral AMD is usually high in iron and sulfate (Kim et al., 2007; Luís et al., 2009). Other AMD contaminants include metals such as: copper, lead, mercury, and zinc. Chronic exposure can result in the bioaccumulation of these contaminants along trophic levels and contribute to the onset of toxicological effects ranging from physiological to reproductive defects (Herlihy et al., 1990; Kim et al., 2007; Ceccatelli et al., 2013; Williams and Turner, 2015).

Two treatment methods are commonly used for AMD remediation: active and passive systems. Overarching goals for both types of systems are to remove heavy metals and sulfate, and for acidic AMD, increase the pH (Tarutis et al., 1992; Johnson and Hallberg, 2002). Passive remediation systems encompass a series of retention ponds, wetlands, and alkaline material to promote the precipitation of heavy metals. Active treatment systems employ continuous addition of active chemicals (i.e., hydrated lime, quick lime, or soda ash) on AMD to forcibly achieve similar results. Although passive systems require periodic maintenance to remove accumulated precipitates, they are not as costly to maintain as active systems.

Since a third of the United States AMD impacted waters originate from Pennsylvania, effective engineering and implementation of AMD remediation systems are integral to solving this long-standing environmental issue (Reinhardt, 1999). There are nearly 300 passive remediation systems in Pennsylvania for which water chemistry and physical parameters have been compiled.^1^ Currently, limited data on the biology of the systems is collected and the performance of these treatment systems is influenced by the types, amounts, and activities of bacteria and aquatic plants (macrophytes) present. To address this gap in research and better understand how microorganisms can contribute to AMD remediation strategies, our study evaluated the spatial distribution of microbial consortia throughout the Middle Branch passive treatment system in Clinton County, Pennsylvania. High-throughput sequencing of the 16S rRNA gene and shotgun metagenomics were employed to investigate aquatic and sediment environments of the remediation system and adjacent receiving stream. Physiochemical parameters were measured to understand its impacts on shaping microbial communities.

## Materials and Methods

### Site Description

Middle Branch is located at 41° 20′ 48.0012″ N, 77° 52′ 3″ W. The system collects acidic mine drainage (pH 2.5–3.5) from both deep and surface mines. Built in 2001, it originally consisted of two vertical flow ponds (called SAPS) in parallel, followed by a settling pond, an aerobic wetland and two aerobic manganese removal beds in parallel that treated two acidic mine discharges. In 2007, renovations were completed to remediate several problems that had developed in the system. After renovation, the one source of acidic mine drainage continues to flow into the system and 60–70% of the second was diverted out of the system. The two vertical flow ponds (SAP1) were refurbished, the settling pond and aerobic wetland remained untouched and the manganese removal beds were converted into vertical flow ponds (SAP2). The AMD in Middle Branch is characterized by low pH, and high levels of aluminum and manganese.^2^ Samples were collected from the input AMD, SAP1, the settling pond, the wetlands, SAP2, upstream and downstream following the system’s discharge from the remediation system (Supplementary Figure S1).

From July 2014 to February 2015, a total of 23 samples were collected from Middle Branch and the passive remediation network in Clinton County, Pennsylvania. Middle Branch was chosen as a test site based on the following criteria: (i) the site utilizes passive remediation system, (ii) the site is easily accessible, and (iii) there are observable changes in water chemistry prior and following the remediation system. Permission for research at these sites was obtained through a research and monitoring agreement between Trout Unlimited and the Pennsylvania Department of Conservation and Natural Resources (DCNR).

### Field Sampling

Samples (*n* = 23) were collected from sediment and water matrices. Sediment samples (*n* = 9) were collected from the center of the stream using sterile 50 mL conical tubes. Approximately 1 cm of sediment was collected per sample. If sediment was excessively firm, sterilized spatulas were used for collection. All sediment samples were placed on ice after collection. Water samples (*n* = 14) were collected in a sterile 1 L Nalgene bottle. The collected water was filtered on site with 0.22 μm polyethersulfone filters (Millipore, Billerica, MA, United States) and subsequently stored in Whirl-Pak bags for future analysis (Nasco, Fort Atkinson, WI, United States). All filtered water samples were put on ice after collection. All samples were stored at −20°C for further processing. The same technique was used for the collection of sediment and water samples at each step of Middle Branch remediation system.

Stream water chemistry measurements (pH, conductivity, temperature) were taken on site at the time of sampling with a PCSTestr 35 Multi-parameter test probe (Oakton Instruments, Vernon Hills, IL, United States) at each sampling location. Water samples at each site were also sent to and analyzed at the Pennsylvania State University Institute for Energy and the Environment Water Quality Lab^3^ to determine the remaining stream water characteristics [sulfate, iron, manganese, aluminum, and total organic carbon (TOC) concentration] following Standard Methods for the Examination of Water and Wastewater, 23rd Edition.

### DNA Extraction, Quantification, 16S rRNA Gene Library Preparation, and Sequencing

For nucleic acid extractions of water samples, filters were removed from each 0.22 μm polyethersulfone filter (Millipore, Billerica, MA, United States) and placed in a MoBio bead beating tube. For nucleic acid extractions of sediment samples, 0.25 g of sediment from each sample was placed in MoBio bead beating tubes (MoBio Laboratories Inc., Carlsbad, CA, United States). All DNA extractions were then performed via the PowerSoil extraction procedure provided by MoBio Laboratories (Carlsbad, CA, United States). DNA extracts were eluted and reconcentrated in 50 ul of 1× TE or sterile water. DNA extracts were quantified using the Qubit 2.0 Fluorometer high sensitivity dsDNA kit, according to the Qubit dsDNA HS Assay Kit protocol (Life Technologies, Grand Island, NY, United States).

Illumina iTag Polymerase Chain Reactions (PCR) were performed in a total volume of 25 μL for each sample and contained final concentrations of 1X PCR buffer, 0.8 mM dNTP’s, 0.625 U Taq, 0.2 μM 515F forward primer, 0.2 μM Illumina 806R reverse barcoded primer and ∼20 ng of template DNA per reaction. PCR was carried out on a MJ Research PTC-200 thermocycler (Bio-Rad, Hercules, CA, United States) using the following cycling conditions: 98°C for 3 min; 35 cycles of 98°C for 1 min, 55°C for 40 s, and 72°C for 1 min; 72°C for 10 min; and kept at 4°C. PCR products were visualized on a 1% SYBRsafe E-gel (Life Technologies, Carlsbad, CA, United States).

Pooled PCR products were gel purified using the Qiagen Gel Purification Kit (Qiagen, Frederick, MD, United States). Clean PCR products were quantified using the Qubit 2.0 Fluorometer (Life Technologies, Carlsbad, CA, United States), and samples were combined in equimolar amounts. Prior to submission for sequencing, libraries were quality checked using the 2100 Bioanalyzer DNA 1000 chip (Agilent Technologies, Santa Clara, CA, United States). Pooled libraries were stored at −20°C until they were shipped on dry ice to the California State University (North Ridge, CA, United States) for sequencing.

Library pools were size verified using the Fragment Analyzer CE (Advanced Analytical Technologies Inc., Ames, IA, United States) and quantified using the Qubit High Sensitivity dsDNA kit (Life Technologies, Carlsbad, CA, United States). After dilution to a final concentration of 1 nM and a 10% spike of PhiX V3 library (Illumina, San Diego, CA, United States), pools were denatured for 5 min in an equal volume of 0.1 N NaOH then further diluted to 12 pM in Illumina’s HT1 buffer. The denatured and PhiX-spiked 12 pM pool was loaded on an Illumina MiSeq V2 300 cycle kit cassette with 16S rRNA library sequencing primers and set for 150 base, paired-end reads.

### Shotgun Metagenomics Library Preparation

Approximately 5–10 ng of DNA per sample (*n* = 10) underwent Nextera (Illumina, San Diego, CA, United States) tagmentation and library preparation and dual index barcoding. Quality of final libraries was assessed using a high sensitivity bioanalyzer chip (Agilent, CA, United States). Equimolar amounts of library were pooled and purified using QIAquick gel purification kit (Qiagen, CA, United States). Purified libraries underwent sequencing on the Illumina HiSeq3000 following a 1 × 100 index run.

### Bioinformatics and Statistical Analysis

Sequences were trimmed at a length of 150 bp and quality filtered at an expected error of less than 0.5% using USEARCH v7 (Edgar, 2013). After quality filtering, reads were analyzed using the Qiime 1.9.0 software package (Caporaso et al., 2010, 2011). Chimeric sequences were identified using USEARCH61 (Edgar, 2010). More than 650,000 sequences for a total of 21 sediment (*n* = 7) and water samples (*n* = 14) were obtained after quality filtering and chimera checking. Open reference operational taxonomic units (OTUs) were picked using the USEARCH61 algorithm (Edgar, 2010), and taxonomy assignment was performed using the Greengenes 16S rRNA gene database (13-5 release, 97%) (DeSantis et al., 2006). Assigned taxonomy were organized into a BIOM formatted OTU table, which was summarized within Qiime-1.9. Bar plots were generated from summarized taxonomy outputs, in which the 8 most abundant phyla were displayed, with all remaining taxa grouped in an “other” category. OTUs that were not classified at the kingdom taxonomic rank were discarded. Heatmaps representing the relative abundance of known sulfate reducing bacteria (SRBs) known to methylate mercury throughout Middle Branch were also generated using summarized taxonomy outputs.

Alpha diversity analysis was conducted within the Qiime 1.9.0 analysis package, to determine the bacterial species richness and evenness within each sample. Alpha rarefaction plots were created at a maximum sequencing depth of 6500 sequences/sample with a step size of 650 sequences/sample for 20 iterations. Rarefactions were then collated and plotted using observed species, Chao1, PD Whole Tree, and Heip’s evenness diversity metrics. BIOM formatted OTU tables underwent CSS normalization for Beta Diversity Analyses. Principal coordinate analysis (PCoA) plots were generated from weighted Unifrac distance matrices (Unifrac, Boulder, CO, United States) within Qiime1.9.0. The PCoA plots were visualized using the R package Plotly. Using the same weighted Unifrac distance matrices, two PCoA plots were generated to visualize how samples plotted against an increasing gradient of sulfate concentration and TOC.

The significance of clustering among samples by sample type (sediment and water) and location were assessed using a weighted Unifrac distance matrix and ANOSIM statistical test within Qiime 1.9.0. Adonis statistical tests were conducted on weighted Unifrac values to assess the magnitude of variation in both sediment and water samples explained by measured environmental parameters (Supplementary Figure S2). Additional Adonis tests were also used to assess the explained variance attributed to sulfate concentration on subsets of the dataset consisting of separated sediment and water samples. Kruskal–Wallis rank sum tests were used to determine significant differences in measured environmental quality data, which were not normally distributed (Supplementary Table S3). Each statistical test was considered significant at α = 0.05. Spearman’s correlations were calculated to compare taxa abundance with continuous abiotic parameters assessed at each sampling location.

LEfSe (Linear discriminant analysis Effect Size) was used to identify significantly enriched taxonomic biomarkers for each sampling site at Middle Branch (Segata et al., 2011). A CSS normalized OTU table was filtered to the genus level and compared by sampling sites at Middle Branch. The two sets of vertical flow ponds failed to exhibit differentially enriched taxonomy as separate categorical values, so they were considered as the same categorical cohort for future LEfSe analysis. Alpha levels of 0.05 were used for both the Kruskal–Wallis and pairwise Wilcoxon tests. Significant features with Linear Discriminant Analysis (LDA) of three were used to determine effect sizes of enriched taxa. These results were then plotted as a cladogram.

The focus of the metagenomic analysis was on key genes for sulfur cycling, to evaluate the effects of AMD passive remediation of sulfate-reducing bacteria. Quality filtering for metagenomic analysis were completed using FastQC reports that determined the quality of the raw metagenomic sequences (Andrews, 2010). Trimmomatic was then utilized to remove reads that did not have a leading phred score of at least 33, trailing phred score of 3, minimum length of 33 nucleotides, and a quality score of at least 4 across a sliding window of nucleotides (Bolger et al., 2014). Unassembled reads were used in the HUMAnN2 (HMP Unified Metabolic Analysis Network) pipeline to profile the presence and absence of microbial pathways and functional genes in for four sediment samples throughout the remediation system and one instream sediment samples from Middle Branch (Abubucker et al., 2012). Within the pipeline, taxonomic profiling was determined using MetaPhlAn 2 (Segata et al., 2012), while functionally annotated species pangenomes were determined using the ChocoPhlAn database. Log transformed reads per kilobase million (RPKM) normalized gene family abundance outputs were used to visualize counts of genes related to sulfur metabolism from the sites at Middle Branch. Genes of interest were visualized through a heatmap and a sulfur metabolism pathway from the Kyoto Encyclopedia of Genes and Genomes (KEGG) database (Kanehisa et al., 2016). The heatmap was made using the R package of “pheatmap” (version, 3.3.2) (Kolde, 2015). The mapped KEGG pathway was generated using the R package of “pathview” (version, 3.3.2) (Luo and Brouwer, 2013).

## Results

### Measured Environmental Parameters

Water chemistry was measured throughout the remediation system and receiving stream included, conductivity, pH, temperature (C), sulfate, TOC, iron, manganese, and aluminum concentrations (Supplementary Table S2). Analytes were filtered for dissolved metals. Nearly all measures, except for TOC, were found to be significantly different between raw AMD, the treatment system, and receiving stream locations (Kruskal–Wallis, *P* ≤ 0.02; Supplementary Table S3). TOC percentages were highest in the first set of vertical flow ponds and settling pond (respectively 36.4 and 22.1%; Supplementary Table S2). Samples collected within the raw AMD discharge and remediation system were shown to have greater sulfate (1208 mg/L), iron (68.5 mg/L), and manganese (27.4 mg/L) concentrations compared to samples collected from the receiving stream (respectively averaging 12.5, 1.75, and 0.15 mg/L; Supplementary Table S2). Sulfate was additionally found to explain a significant amount of variation in microbial community structure when separately analyzing sediment (52.8%, Adonis *P* < 0.002) and water (43.2%, Adonis *P* < 0.003) samples.

Of the measured parameters, considering both water and sediment samples Adonis tests yielded significant amounts of variation in microbial communities, which can be attributed to temperature, aluminum, and TOC concentrations (48.6, 47.9, and 45.8%, respectively Adonis *P* ≤ 0.002). Sulfate, iron, and manganese concentrations were found to contribute a smaller magnitude of variation among microbial communities (22.15, 21.96, and 21.83%, respectively Adonis *P* ≤ 0.002).

### Bacterial Community Diversity

**FIGURE 1:**
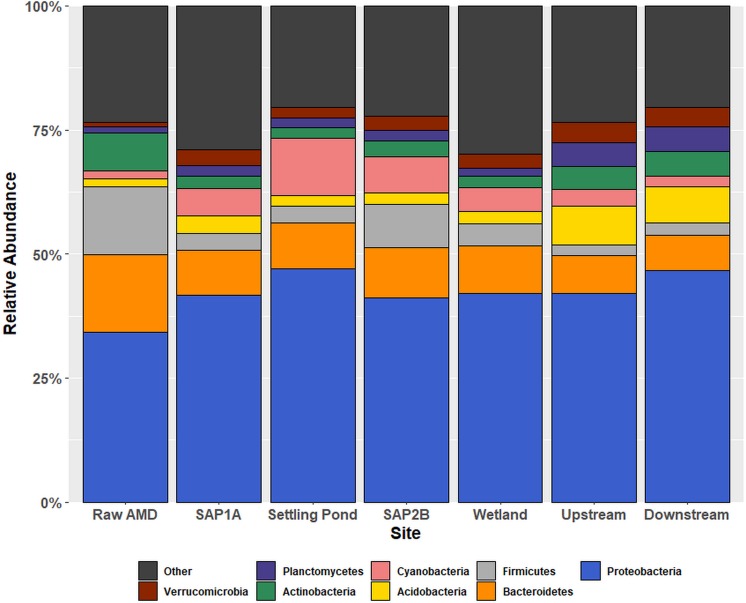
Relative abundance bar chart of OTUs at the phylum taxonomic rank within each sampling location. Relative abundance outputs were generated from an unrarified OTU table picked using the USEARCH sequence analysis tool. The *x*-axis displays all sampling locations in chronological order, and the *y*-axis represents phyla abundance. The eight phyla that constituted the greatest percentage of total sample community composition across all sample locations within each sample matrix are plotted, and all remaining phyla were grouped into the “other” category.

Alpha diversity analysis of 16S rRNA gene sequences revealed significant differences in species richness between sites (*P* < 0.05; Supplementary Table S1). In addition, observed shifts at the phylum-level of the bacterial community structure were based on location throughout Middle Branch (Figure 1). Raw AMD waste samples exhibited an elevated relative abundance of Firmicutes (13.6%) compared to all other sampling locations (average 4%). Conversely, sequences matching the Proteobacteria comprised a greater portion (average 43%) of the bacterial community within and downstream of the passive remediation system compared to raw samples (34%). The top 10 most relatively abundant orders of Proteobacteria diversity showed to range from *Burkholderiales* and *Rhizobiales* with the highest relative abundances to *Rhodobacterales* and *Bdellovibrionales* with the lowest relative abundances across sites (Supplementary Figure S4).

**FIGURE 2:**
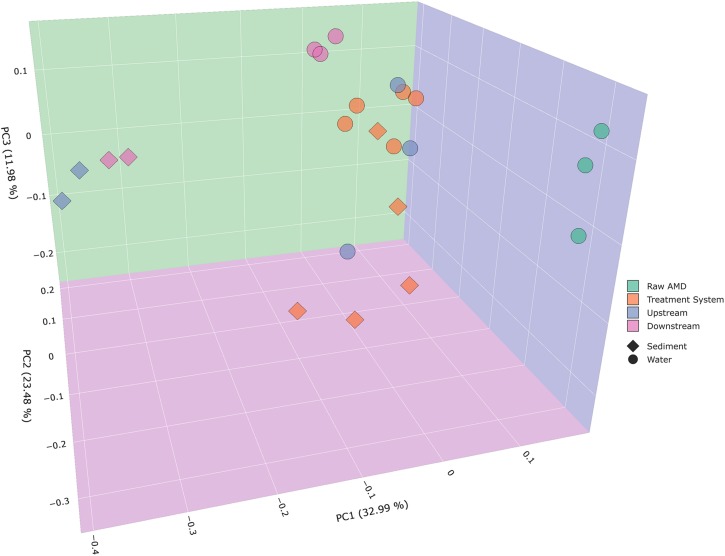
Principal coordinates analysis (PCoA) plots were used to visualize differences in weighted Unifrac distances of instream, treatment ponds, and raw AMD input samples. Points clustered closely together are similar in terms of phylogenetic distance, whereas points that are distant from each other are phylogenetically distinct. Communities from the raw input from a distinct cluster from the rest of the sites, which include upstream, downstream, and the treatment system (ANOSIM *P* = 0.001). As indicated by the figure legend, points were colored by site (raw AMD, treatment system, upstream, and downstream) and shaped based on sample type (sediment and water).

**FIGURE 3:**
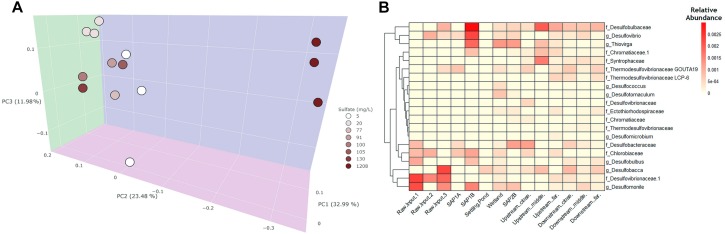
A weighted principal coordinates axes (PCoA) plot and heatmap were generated to visualize community differences between water samples throughout Middle Branch. **(A)** The PcoA plot exhibits water samples which are colored based on sulfate concentration. Sites within the system and the receiving stream are clustered together toward the left. The raw AMD samples are highest in sulfate concentration and distinctly cluster separately from the treatment system and receiving stream. **(B)** Heatmap of SRB and SOB relative abundance throughout the remediation system. Darker shades of red indicate higher relative abundance whereas, lighter shades of color indicate lower relative abundance.

Beta diversity analysis of bacterial communities displayed significantly distinct microbial community compositions between samples collected throughout the system and receiving stream. Weighted PCoA plots exhibited distinct differences in bacterial composition with respect to the type of samples collected in sediment and water (ANOSIM *P* = 0.001). For instance, there is a distinct cluster of upstream and downstream water samples that are separated from sediment samples collected from the same instream location. Sediment samples resulted to cluster closer toward sediment samples from the treatment system. Bacterial communities in raw AMD samples formed a distinct cluster along the PC1 (principal coordinate 1) axis, while samples from the treatment system and receiving stream were located along the PC2 axis (Figure 2). A total of 32.99% variation in the bacterial community can be explained by PC1, suggesting that location within the treatment facility and stream have strong roles in shaping bacterial community structure in both water and sediment samples. The PCoA plots permit for the visualization of bacterial community differences between samples across varying sulfate concentrations (Figure 3A and Supplementary Figure S3A). As sulfate concentrations decreased throughout the progression of the system (Supplementary Table S2), beta diversity of bacterial communities uniquely clustered along the decreasing sulfur gradient in both sample types collected (Figure 3A and Supplementary Figure S3A).

**FIGURE 4:**
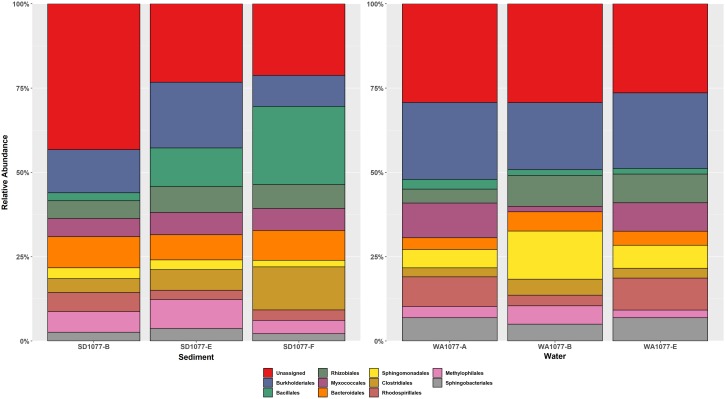
Relative abundance bar graphs of the top 10 OTUs at the order taxonomic rank within SAPS locations. The *x*-axis displays all sampling locations in chronological order, and the *y*-axis represents order abundance. The top 10 taxonomic order constituted the greatest percentage of total sample community composition across SAPS samples. Sample type (water and sediment) were separated into unique 100% bar graphs, where sediment samples are displayed on the right and water samples on the left half of the figure.

Further analysis of paired communities in sediment and water samples from each site were considered. Despite most sites lacked the water and sediment sample size necessary for statistical power, combined samples from both sets of SAPS were an exception with paired groups of three samples each. Statistical ADONIS analysis of paired communities between water and sediment SAPS samples suggest minimal bacterial community differences (ADONIS *P* = 0.369). Alpha diversity metrics revealed no statistically significant differences in species richness between water and sediment samples from SAPS locations (*P* > 0.05; Supplementary Table S4). This lack of bacterial community differences is further reinforced through similarities in relative abundance of the top 10 most abundant taxonomic order (Figure 4).

While trends from beta diversity analysis revealed shifts in bacterial community structure by location and sulfate concentration, further analysis using LEfSe highlighted the distribution of enriched bacterial lineages associated with raw AMD discharge, within the passive remediation system, and the receiving stream (Supplementary Figure S5). Locations within the remediation system were shown to be enriched by fewer bacterial taxa in comparison to the raw AMD discharge (27 taxa) and the receiving stream (upstream: 10 enriched taxa and downstream: 19 enriched taxa) (LDA cutoff score of 3 and *P* < 0.05). The aerobic wetland was dominated by Elusimicrobia, while the settling pond had an enrichment of six bacterial taxa, all belonging to the Cyanobacteria. The raw AMD discharge and vertical flow ponds were the only sites that significantly enriched Firmicutes (Clostridia and Bacilli) (Supplementary Figure S5).

### Observable Shifts in Relative Abundance of SRB and SOB Communities

Since sulfate concentration explained the most variation in bacterial community structure across sediment samples (Supplementary Figure S3A), the relative abundance of sulfur metabolizing (i.e., sulfate reducing and sulfur oxidizing) bacteria were further investigated regardless of their overall low relative abundance among the bacterial community. SRB and sulfur oxidizing (SOB) bacterial assemblages were shown to be more abundant in sediment (average abundance 2%; Supplementary Figure S3B) environments as compared to the water samples (average abundance <1%; Figure 3B). Additionally, sediment samples revealed that the remediation system contained an elevated abundance of SOB and SRB taxa (average abundance 2%; Supplementary Figure S3B) compared to the receiving stream and across all water samples (average abundance <1%; Figure 3B). This trend was also observed from a dendrogram that highlighted phylogenetic distributions of SRB and SOB taxa throughout the system (Supplementary Figure S6). SRBs were predominantly enriched in the remediation system and receiving stream, while lower relative abundances of SOBs were found across all sites. Sediment from the settling pond contained trace amounts of SRBs and SOBs (relative abundance 0.5%; Supplementary Figure S3B), while water sampled from the same site lacked SRBs and SOBs overall. Raw AMD samples were largely enriched with Firmicutes, several members of which are known to contribute to the generation of AMD (Supplementary Figure S5).

The three most abundant taxa among all sulfate reducers in both sediment and water samples were: *Desulfobulbaceae*, *Desulfobacteraceae*, and *Desulfomonile* OTUs. Additionally, the three most abundant sulfur oxidizers included, *Thiovirga*, *Chlorobiaceae*, and *Chromatiaceae*. Elevated relative abundance of these taxa was observed within the sediment samples (average abundance 0.011%; Supplementary Figure S3B), rather than water samples (average abundance 0.005%; Figure 3B). These taxa were of significant interest to this study because they are known to contribute to sulfur cycling activity and can ultimately help in recognizing which bacterial taxa are potentially driving sulfur cycling at this remediation system.

### Shotgun Metagenomic Profiles of Middle Branch Sediments

**FIGURE 5:**
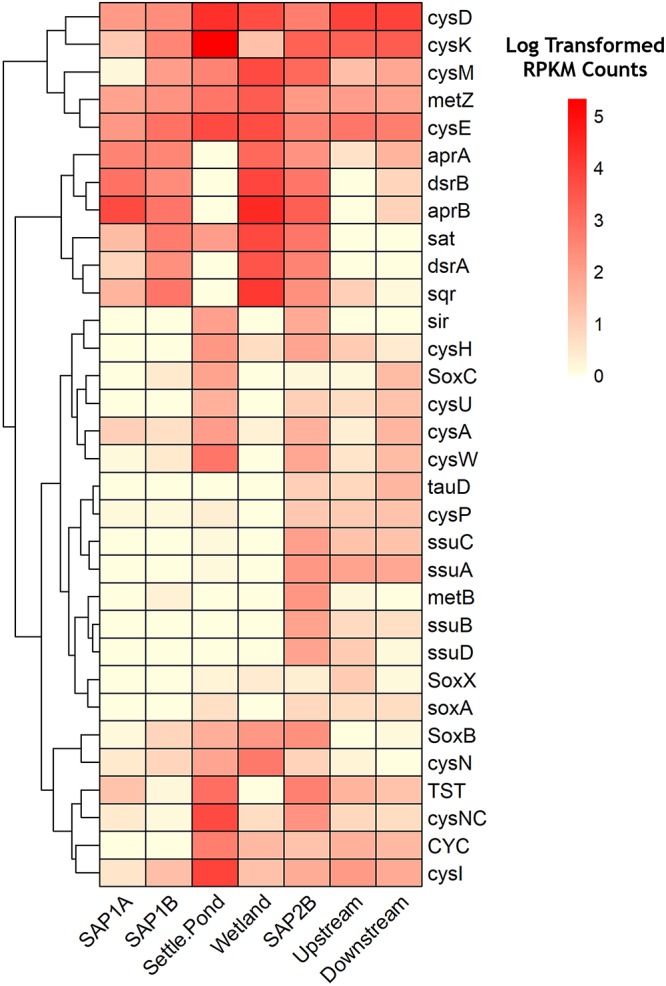
Heatmap of relative abundance levels of genes (KO numbers) related to sulfur metabolism throughout Middle Branch remediation system. Darker shades of red represent a higher level of abundance while lighter shades signify lower expression levels. Clustering along the top of the heatmap represent genes associated with dissimilatory sulfate reduction/oxidation. Clustering at the bottom represent genes associated with assimilatory sulfate reduction/oxidation. Genes with log transformed RPKM Counts below 1.5 were removed from the heatmap.

Metagenomic analysis revealed the relative abundances of key functional genes involved in sulfur metabolism, permitting a greater understanding of how microorganisms could be contributing to the remediation process via sulfur transformations (Figure 5 and Supplementary Figure S7). Dissimilatory sulfate reduction and oxidation pathways were shown to have the highest log transformed RPKM counts throughout sediments from the remediation system (Figure 5). Increased abundance of *aprA* and *aprB* genes from sediments of the remediation system as compared to instream samples support the 16S rRNA results that exhibited higher relative abundance of sulfur metabolizing bacterial assemblages at the same locations (Figure 5). These two genes code for subunits of adenylylsulfate reductase, an integral enzyme utilized in dissimilatory sulfate reduction pathways (Bradley et al., 2011). Genes involved in other sulfur oxidation pathways (e.g., *sox* pathway) exhibited lower log transformed counts (average abundance <1%) across all samples (Figure 5). However, all locations were observed to have at least seven times more *sox* gene counts compared to the vertical flow pond (SAP1A). Both sets of vertical flow ponds and the aerobic wetland were 1.5 times more abundant in assimilatory and dissimilatory sulfate reduction genes compared to the receiving stream.

## Discussion

High-throughput sequencing of the 16S rRNA gene permitted for an in-depth analysis of diverse aquatic and sediment microbial communities within the Middle Branch passive remediation system in Clinton County, Pennsylvania. We examined shifts in bacterial community structure and their expressed genes within a multi-staged passive AMD remediation system and its adjacent receiving stream. Bacterial community structure differentiated by stages of the treatment system, as well as with abiotic parameters such as TOC (45.8%, Adonis *P* = 0.005), sulfate (22.1%, Adonis *P* = 0.001), iron (22%, Adonis *P* = 0.005), and manganese (22%, Adonis *P* = 0.005) concentrations. Concentrations of these environmental parameters significantly explain a degree of variation in the microbial community profile throughout the remediation system. This study permits an in depth understanding of how biotic and abiotic factors interact within the varying remediation structures of the overall system. These findings are also informative to environmental engineers for future projects as it identified relationships between bacteria, macrophyte, and physiochemical factors that contribute to the efficiency and sustainability of Middle Branch as remediation system and similar passive treatment systems.

### Distinct Acidophilic Bacterial Communities of Raw AMD Discharge

Alpha and beta diversity analysis of bacterial communities associated with raw AMD discharge were found to contain significantly less observed species (*p* < 0.05) and phylogenetically distinct bacterial communities as compared to those in the receiving stream (Figure 1 and Supplementary Table S1). Raw AMD discharge water contained a higher relative abundance of Firmicutes compared to other locations, supporting previous studies that suggest the ability of acidophilic bacteria to thrive in contaminated AMD ecosystems (Church et al., 2007; Alazard et al., 2010; Chodak et al., 2013; Holanda et al., 2016). Acidimicrobiales, *Methylophilaceae*, *Legionellaceae* were enriched in raw AMD samples, all of which have also been found to thrive in AMD environments (Supplementary Figure S5) (Rastogi et al., 2010; Yang et al., 2014; Bier et al., 2015). Increased relative abundance of SRBs were additionally found within raw AMD discharge and the remediation system, rather than the instream sites (Figure 3B and Supplementary Figures S3B, S5), indicating that raw AMD environments are not only capable of enriching bacterial lineages that generate AMD, but also SRBs (Muyzer and Stams, 2008). This is consistent with other studies that have isolated acid-tolerant SRBs from acidic mine drainage (Rowe et al., 2007; Sánchez-Andrea et al., 2012). In varying circumstances, others have found that limestone and other materials to likely enrich for a diversity of SRBs (Beffa et al., 1996; Marques et al., 2019). Similarly, these materials are included within most passive remediation systems, including Middle Branch, and could be contributing sources of observed SRBs in these engineered systems. The ability for AMD contaminated environments to selectively enrich for SRBs is important to consider for several reasons including the fact that these communities can produce soluble sulfide compounds within raw AMD waste and remediation structures (i.e., vertical flow ponds, aerobic wetland) alike.

### Microbially Driven Sulfur Cycling Among Vertical Layers of SAPS

Successive alkalinity producing ponds (SAPS) also known as vertical flow ponds (VFPS) are layered structures commonly used in passive remediation systems with acidic AMD. SAPS are ultimately designed to transform ferric iron to its reduced form, ferrous iron, and facilitate an anoxic/alkaline environment through the organic matter and limestone layers. These facilitate an environment that’s not only ideal for the reduction of sulfate but also the precipitation of metal hydroxides and sulfides. Shifts in microbial community structure from raw AMD to those of the treatment system could be attributed to the physiochemical changes of TOC (total organic carbon) and sulfate content occurring within the SAPS (Figure 3A and Supplementary Figures S3A, S8, and Supplementary Table S2). High concentrations of TOC in SAPS correspond with physicochemical properties of organic matter layers integrated within these remediation structures (Cheong et al., 2012). Carbon limitation can also be a regulator of sulfate reduction in acidic mining lakes (Blodau, 2006), explaining the increase in SRB activity with higher TOC. Additionally, the relatively low iron levels found in the system (<2 mg/L) provides a greater opportunity for sulfate reduction, as other studies involving similar physicochemical properties and low pH (2.6) at Mining Lake 111 found that iron reduction was stimulated before sulfate reduction (Wendt-Potthoff et al., 2002). This aligns well with our observation of lower concentrations of sulfate in the SAPS, indicating these structures are successfully enriching for microbes that can catalyze sulfur redox transformations (Church et al., 2007; Muyzer and Stams, 2008).

Relative abundance of both SRBs and SOBs were highest within the SAPS and aerobic wetland, suggesting that higher rates of sulfur redox transformations are likely to occur within the interface of aerobic and anaerobic sediment environments within these remediation structures. It has been shown that SOBs residing in an organic carbon-rich permeable reactive barrier could be oxidizing the reduced sulfur species that SRBs were producing, through complete and incomplete reduction, thus creating an efficient redox environment between the SOBs and SRBs (Benner et al., 2000). The structural layering of SAPS could be shaping a microbial community that’s capable of maintaining a similar redox environment as previously described by Benner et al. (2000) with permeable reactive barriers. There also appears to be a potential for anaerobic phototrophic sulfur oxidation occurring in the SAPS, as our study revealed enrichment of taxa belonging to *Chlorobiaceae* and *Chromatiaceae* (Figure 3B and Supplementary Figure S3B), both of which contain phototrophic SOBs (*Chlorobium* and *Allochromatium*, respectively) (Madigan et al., 2005). The combination of increased TOC and the presence of sulfate in the SAPS of Middle Branch imply how integral these structures are to the efficiency of passive remediation systems because these factors are favorable conditions for organotrophic bacteria like SRBs (Jung et al., 2014).

Metagenomic profiling allowed for further understanding of the functional capabilities of the microbial communities. There are three types of sulfur metabolism: assimilatory, dissimilatory, and *sox* mediated. The sulfate assimilatory pathway yields reduced sulfur compounds for the biosynthesis of certain amino acids. Rather than the incorporation of sulfur compounds, dissimilatory reduction of sulfate results in the excretion of sulfide and is commonly used for bacterial anaerobic respiration. The *sox* mediated pathway yields oxidized sulfur species and is widely used among phototrophs and chemolithoautotrophic bacteria. These sulfur metabolisms are important to consider in regard to this study because each pathway plays a major part of the biogeochemical sulfur cycle. SAPS contained higher relative abundance of the gene homologs *aprAB* and *dsrAB* which encode for adenylylsulfate reductase and dissimilatory sulfite reductase, key enzymes associated with sulfate reduction processes. These enzymes are thought to be the rate-limiting step of SRB-mediated sulfate reduction under conditions of varying sources of organic matter (Zhang et al., 2017). Both sets of vertical flow ponds in the remediation system contained these gene homologs at similar levels of abundance, suggesting the microbial communities’ capability in reducing sulfate. The high prevalence of sulfide/quinone oxidoreductase (*sqr*) in the SAPS suggests sulfur oxidation by way of a sulfite intermediate, rather than direct oxidation to sulfate (i.e., *sox*). This is further enhanced in the first set of SAPS where lower relative abundance of sulfur oxidation (*sox*) genes were found, compared to the last set of SAPS (Figure 5).

### Accumulation of Insoluble Sulfates and Sulfur Assimilation in Middle Branch Settling Pond

Cyanobacteria were the only significantly enriched phyla within the settling pond (Supplementary Figure S5) and were identified as orders of previously identified oxygenic photosynthetic blue-green algae: Chlorophyta, Stramenopiles, and Streptophyta (Leipe et al., 1996). The enrichment of Cyanobacteria within the oxidation/settling pond at Middle Branch aligns well with findings from another study where microbial communities in an oxidation pond were also dominated by Cyanobacteria (Phillips et al., 1995). SRBs and SOBs are likely to not be present within the settling pond because it is a highly oxic environment, where it is mechanistically designed to naturally oxidize heavy metals into insoluble solids. The lack in abundance of microbial communities and key gene homologs that transform sulfur in the settling pond either suggest that SRBs and SOBs are not driving sulfur cycling, or microbial-driven sulfur cycling isn’t occurring, and the settling pond is accruing metal sulfides from the first set of SAPS (sulfate concentration ∼30 mg/L more in settling pond than SAP1).

Increased TOC concentrations in the settling pond are likely due to the enriched Cyanobacteria because they photosynthetically produce dissolved organic carbon and extracellular organic matter (Fallowfield and Daft, 1988). Additionally, increased sulfate concentrations could be a result of the settling pond receiving output from two parallel SAPS at the beginning of the system where they may be overwhelmed by incoming AMD and not able to completely reduce all sulfate. With inflow coming from two SAPS and a design engineered to control retention time of water in the pond, a resulting increase of sulfate indicates a possible bottleneck in the system where forms of sulfur compounds are accumulating.

Despite a low abundance of sulfur utilizing bacteria and the genes involved in the dissimilatory sulfate reduction pathway in the settling pond (Figure 3B and Supplementary Figures S3B, S5), the genes involved in sulfur assimilation were enriched in this environment. Specifically, those involved in cysteine production by way of the *cysJIHDNG* operon (Figure 5). Cysteine production occurs through intermediates found in sulfur cycling (APS, sulfite, and sulfide) (Méndez-García et al., 2015). Elevated levels of *cysD* and *cysN*, genes that in many organisms code for ATP sulfurylase (Leyh et al., 1988), is the first step in sulfur assimilation which leads to the production of APS from sulfate. The high relative abundance of *cysH*, an APS reductase, in the settling pond, suggests the bacterial consortia therein were capable reducing APS to sulfite. It should be noted that *cysH* has been found in acidic sulfur bacteria such as the extremophile *A. ferrooxidans* (Valdés et al., 2003). The next step in sulfur assimilation is the reduction of sulfite to sulfide, catalyzed by the enzyme sulfite reductase and consists of two subunits, an alpha (encoded by *cysJ*) and a beta (encoded by *cysI*) (Kredich and Tomkins, 1966). The *cysI* (beta subunit), *cysM* and *cysE* genes were enriched in the settling pond (Figure 5), and these genes are involved in the final steps of sulfur assimilation (Kredich and Tomkins, 1966; Valdés et al., 2003). Oxygenic organisms, including cyanobacteria, have been recognized to assimilate reduced sulfate compounds for protein, amino acid, and sulfolipid biosynthesis (Schmidt, 1988). This ultimately suggests that the cyanobacteria in the settling pond could be highly capable in assimilating reduced sulfate, rather than dissimilatory sulfate reduction. Overall, our data showed an increase in the sulfur assimilation pathway occurring in the settling pond of Middle Branch.

### Potential for Microbially Driven Sulfur Cycling Between Anaerobic and Aerobic Interfaces of Wetland

The highest abundance of sulfur transforming bacterial communities and essential genes for dissimilatory sulfate reduction were primarily associated within the SAPS and the aerobic wetland (Figure 3B and Supplementary Figures S3B, S5). Sulfate reducing communities largely dominated wetland sediments, rather than water samples because sediments are a more anaerobic environment than the combined water and macrophyte layer above. Water samples, in this study, were largely dominated by photoautotrophic (*Chlorobiaceae*) and sulfur-oxidizing bacteria (*Thiovirga*), as previously noted in other studies (Pfennig and Trüper, 1981; Ito et al., 2005) (Figure 3B). Chemolithoautotrophic SOBs like *Thiovirga sulfuroxydans*, can grow on reduced sulfur compounds (thiosulfate, elemental sulfur, and sulfide), all of which are part of the sulfur redox environment (Ito et al., 2005).

Communities associated with the aerobic and anaerobic interfaces of the SAPS, including the aerobic wetland, were found to be functionally capable of promoting a sulfur redox environment (Figure 3B and Supplementary Figure S3B). Emphasis of these oxic interfaces of constructed wetlands to remove manganese and arsenic have been previously identified, further supporting our findings which imply that microbially mediated sulfur redox environments could contributing to AMD remediation processes (Clayton et al., 1997; Bednar et al., 2005). Others have also found that organic rich layers in aerobic wetland are integral to providing substrate for the proliferation vegetation that absorb metals and control water velocity (Tarutis and Unz, 1995; Nicomrat et al., 2006).

### Metabolically Diverse Microorganisms in Receiving Streams Following AMD Remediation

Alpha and beta diversity analysis revealed that the receiving stream had the highest observed species and phylogenetically distinct communities compared to the raw AMD input (Figure 2 and Supplementary Table S1). Parallel to our findings, previous work that characterized the taxonomic and functional differences of microbial communities between Qinghai Lake and input streams found that taxonomic and functional diversity from input streams were significantly greater than those from Qinghai Lake (Johnson, 2016; Ren et al., 2017). Although their study didn’t explore the impacts of AMD and its remediation on receiving streams, it supports our observations that receiving streams are generally more diverse than other environments when nutrient availability is highly specific to the site (Johnson, 2016; Ren et al., 2017). Our study showed that the receiving stream had a low abundance of sulfur transforming bacteria and essential gene homologs. Although its implied that the receiving stream doesn’t cycle sulfur in conventional pathways of interest to this study, other integral nutrient cycling communities are present like nitrogen fixers and carbon cycling bacteria (Supplementary Figure S5) (Kuever, 2014; Brewer et al., 2016; Hermans et al., 2017). Others have noted the importance of diverse stream microorganisms in the biogeochemical cycling of organic matter, trophic transfer of nutrients, and the biotransformation of contaminants (Battin et al., 2003; Von et al., 2007; Findlay, 2010; Robert et al., 2015).

## Conclusion

While many have characterized the microbial community structure and their functional roles in the generation of raw AMD, passive remediation systems have been largely understudied. Considering this gap of knowledge, this study identified several taxonomic lineages capable of facilitating sulfur redox environments within passive remediation structures of Middle Branch. Additionally, TOC and sulfate concentration influenced bacterial community structure and associated metabolic capabilities in remediation structures including vertical flow ponds and aerobic wetlands; further contributing to our understanding of engineered remediation structures and its contribution to the microbially mediated fraction of the remediation process. Further implications of this study include establishing a groundwork for utilizing meta-omics approaches to improve the development of efficient and robust treatment of AMD waste.

Future work should investigate microbial communities’ structure and chemistry in several different passive remediation systems. More research into the biotic and abiotic parameters that govern significant microbial dynamics within these passive remediation systems have larger implications for environmental engineers and scientists who design remediation systems. Spatial and seasonal variations between different passive remediation systems should also be considered in future studies, as these variables may play significant roles in shaping distinct microbial communities in remediation systems.

## Author Contributions

RL, SR, and CG designed the study. TL, NW CM, and NU collected and processed the samples for sequencing. TL, JW, NW, and RL wrote the manuscript. All authors were involved in data analyses, interpretation of data, figures and table generation for the manuscript, and read and revised the manuscript.

## Conflict of Interest Statement

JW was employed by company Wright Labs, LLC. The remaining authors declare that the research was conducted in the absence of any commercial or financial relationships that could be construed as a potential conflict of interest.

## Footnotes

1 https://www.datashed.org/

2 https://www.datashed.org/middle-branch-two-mile-run/

3 http://eesl.iee.psu.edu/content/water-quality-laboratory
