# Supplementary material for: Microbial Communities Associated With Passive Acidic Abandoned Coal Mine Remediation
Source: Front Microbiol. 2019 Aug 23;10:1955. doi: 10.3389/fmicb.2019.01955 (PMC6716070; doi:10.3389/fmicb.2019.01955)
Supplement: Supplementary file 1 [file Data_Sheet_1.docx]

Supplementary Material

Microbial Communities Associated with Passive Acidic Abandoned Coal Mine Remediation

Truc Ly^1^, Justin R. Wright^2^, Nicholas Weit^1^, Christopher J. McLimans^1^, Nikea Ulrich^1^, Vasily Tokarev^2^, Michelle M. Valkanas^3^, Nancy Trun^3^, Shawn Rummel^4^, Christopher J. Grant^1^, Regina Lamendella^1,2^

^1^Juniata College, Department of Biology, Huntingdon, 16652, USA

^2^Wright Labs LLC., Huntingdon, PA 16652, USA

^3^Duquesne University, Department of Biological Sciences, Pittsburgh, PA 15282, USA

^4^Trout Unlimited, Arlington, VA, 22209, USA

*** Correspondence:** Corresponding Author: Regina Lamendella

lamendella@juniata.edu


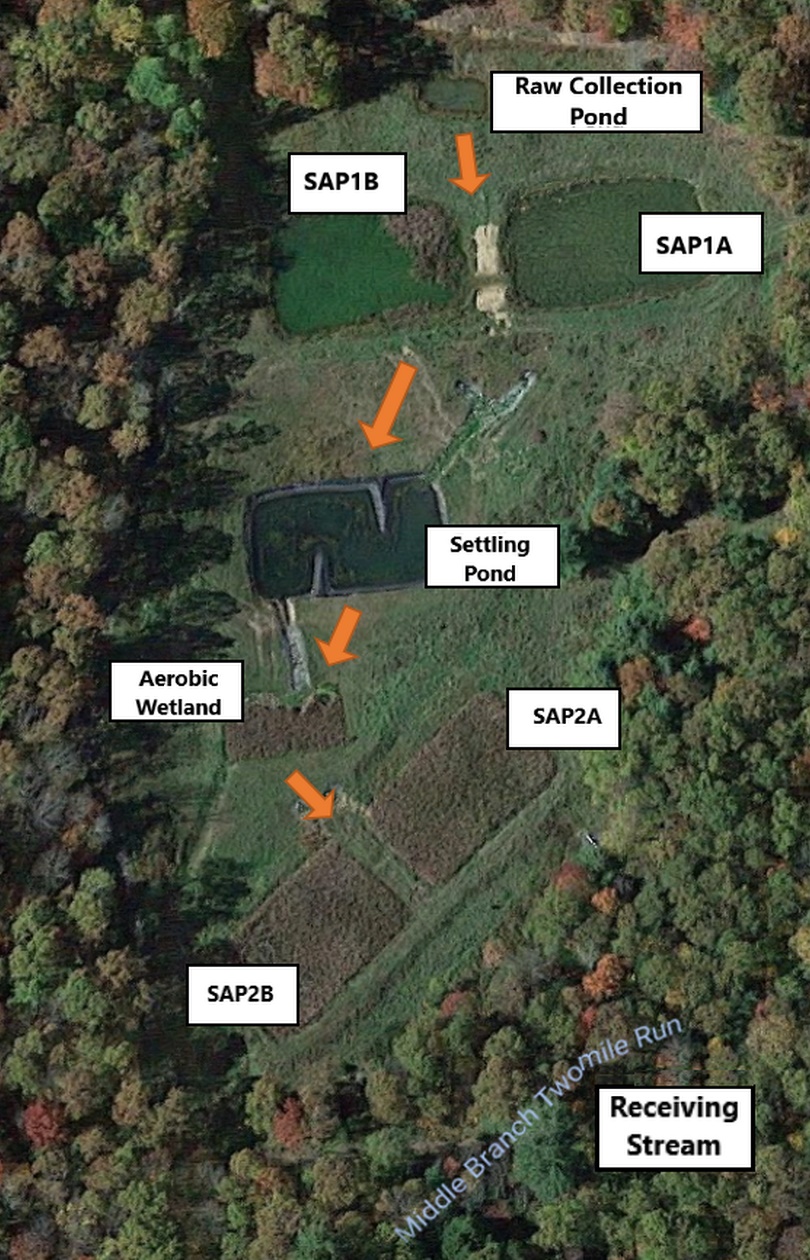


**Figure S1:** Map viewing locations throughout Middle Branch passive remediation system at Clinton County, Pennsylvania. The remediation system contains two sets of successive alkalinity producing systems (SAPS) also known as vertical flow ponds (VPS), sedimentation pond, and an aerobic wetland.

**Figure S2:** Bar graph exhibiting Adonis statistical test results across sediment and water samples throughout the remediation system and receiving stream.

**Table S1:** Table comparing bacterial richness using alpha diversity metrics. Alpha diversity was calculated for each site: raw AMD discharge, treatment (all sites within the system), and instream. Alpha diversity measures revealed significant differences in species richness between all sites (P < 0.05) except between treatment and raw AMD discharge sites.

| **Location** | **Alpha Diversity Metrics** | | | |
| --- | --- | --- | --- | --- |
|  | Observed | Chao1 | heipE | PTDTree |
| Raw AMD Input  N = 3 | 449 ± 101 | 852 ± 175 | 0.165 ± 0.078 | 56.7 ± 11.3 |
| Treatment  N = 10 | 555 ± 215 | 1350 ± 509 | 0.368 ± 0.191 | 60.9 ± 22.5 |
| Instream  N = 10 | 977 ± 106 | 2630 ± 520 | 0.691 ± 0.082 | 92.6 ± 7.82 |

**Table S2**: Measured environmental parameters throughout Middle Branch remediation system

and its receiving stream.

| **Location** | **Properties** | | | | | | | |
| --- | --- | --- | --- | --- | --- | --- | --- | --- |
|  | Sulfate (mg/L) | TOC (%) | Fe (mg/L) | Mn (mg/L) | Al (mg/L) | Lab pH | Conductivity (Umhos/cm) | Temperature (Cº) |
| Raw AMD | 1208 | NA | 68.45 | 27.36 | NA | 3 | 1650 | 0.7 |
| SAP1A/SAP1B | 91/77 | 36.36 | 0.25/0.23 | 1.54/1.48 | 0.1/0.05 | 7.6 | 355/325 | 12 |
| Wetland | 105 | NA | 0 | 2.5 | 1.04 | 5.1 | 255 | 15.8 |
| Settling Pond | 130 | 22.05 | 0.14 | 3.67 | 1.68 | 5.3 | 300 | 18.9 |
| SAP2B | 100 | 17.6 | 0.05 | 0.37 | 0 | 7.3 | 347 | 13.1 |
| Upstream | 5 | 1.03 | 0 | 0 | 0.1 | 5.7 | 31 | 11.1 |
| Downstream | 20 | 1.44 | 1.75 | 0.15 | 2.49 | 7.5 | 108 | 12.1 |

**Table S3:** Kruskal Wallis rank sum correlations between locations (raw AMD, treatment system, upstream, and downstream) and environmental parameters

| **Properties** | **Kruskal Wallis p-values** |
| --- | --- |
| Sulfate (mg/L) | 0.00015 |
| TOC (%) | 0.55270 |
| Fe (mg/L) | 0.00026 |
| Mn (mg/L) | 0.00015 |
| Al (mg/L) | 0.00102 |
| pH in Lab | 0.02282 |
| Conductivity (Umhos/cm) | 0.00015 |
| Temperature (Cº) | 0.00121 |


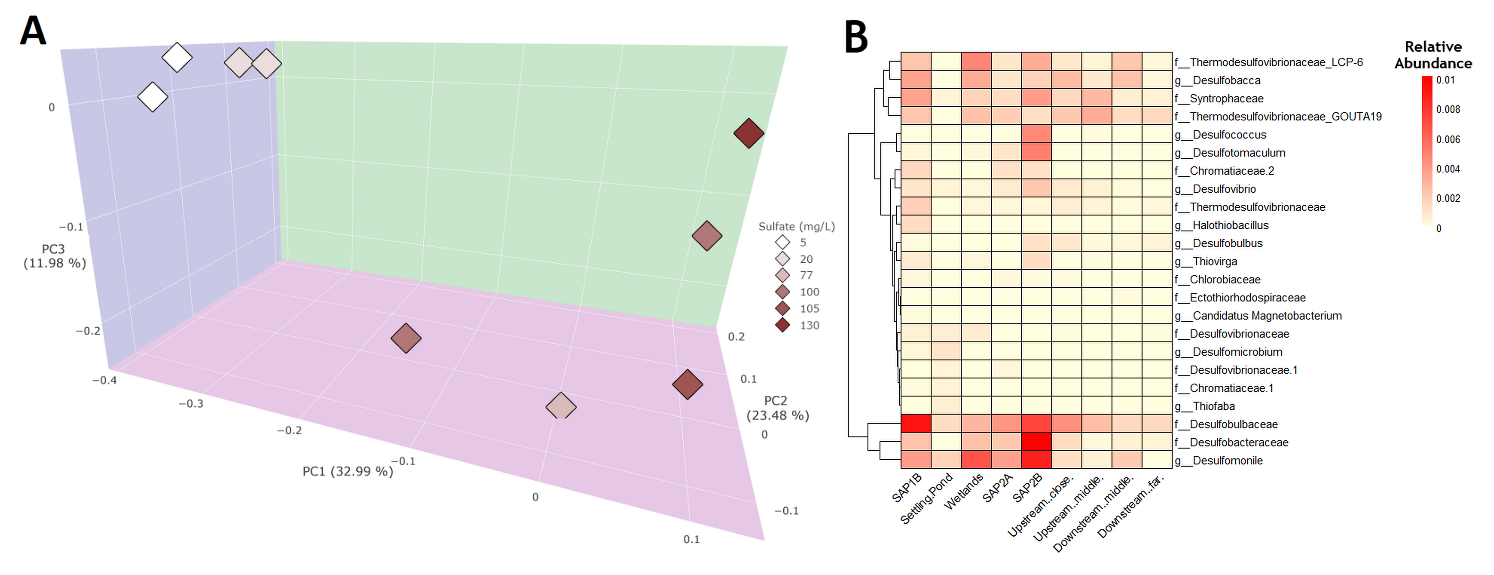


**Figure S3:** A weighted principal coordinates axes (PCoA) plot and heatmap were generated to visualize community differences between sediment samples throughout Middle Branch. (A) The PcoA plot exhibits sediment samples which are colored based on sulfate concentration. Sulfate was found to cause the greatest amount of variation in microbial community structure between sediment samples (52.8%, Adonis P <0.002). Sites within the system (right cluster of diamonds) were not only found to have distinctly different microbial community structures from those from the receiving stream (left cluster of diamonds), but also different sulfate concentrations. (B) Heatmap of SRBs and SOBs relative abundance throughout the remediation system. Darker shades of red indicate higher relative abundance whereas, lighter shades of color indicate lower relative abundance.


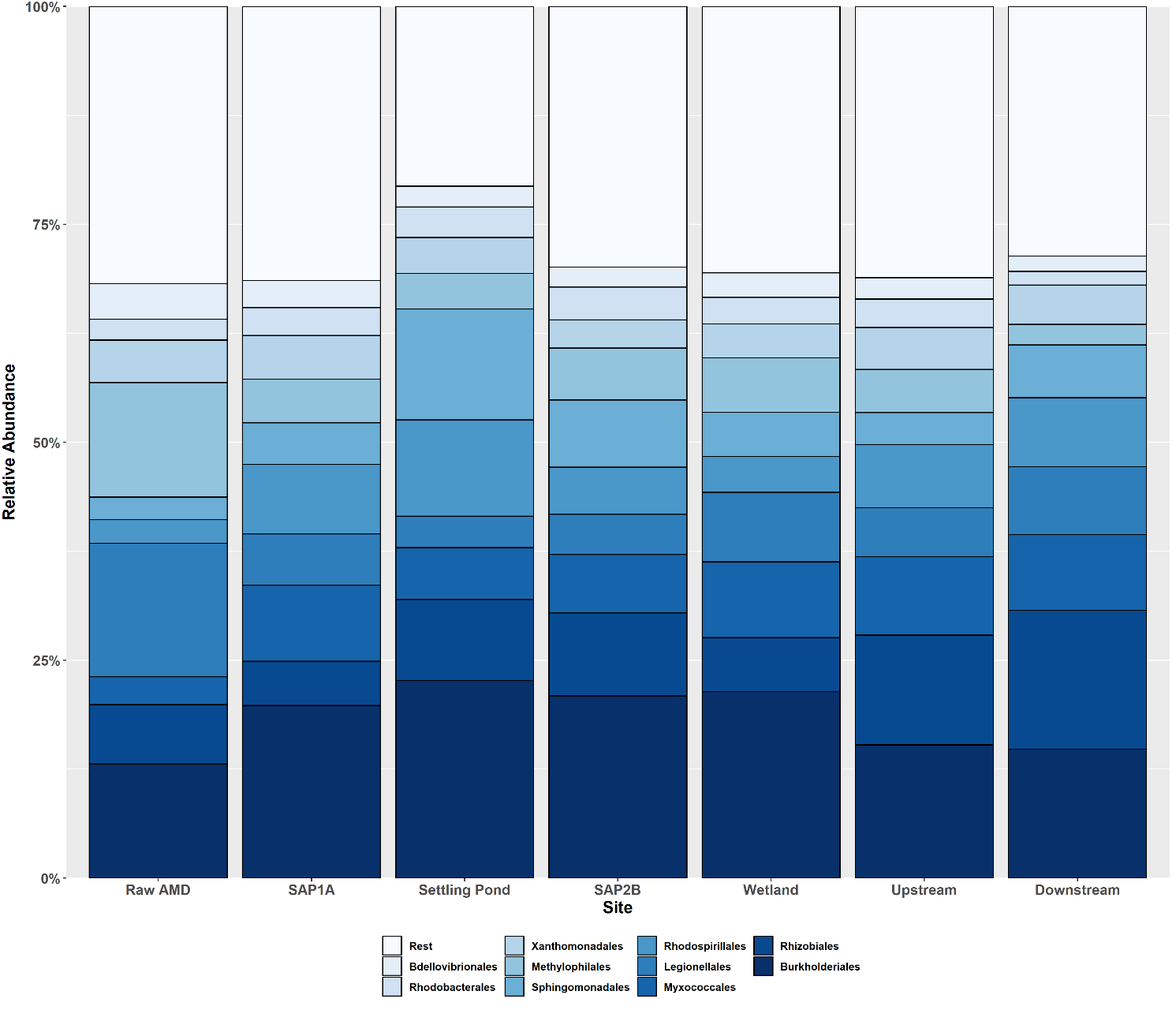


**Figure S4:** 100% bar plot of top 10 order of bacteria across sites throughout remediation system. This was generated using an unrarified OTU table picked using the USEARCH sequence analysis tool. The x-axis displays all sampling locations in chronological order, and the y-axis represents order abundance. The top 10 order of bacteria constituted the greatest percentage of total sample community composition across all sites. All remaining order of bacteria were grouped into the ‘other’ category.


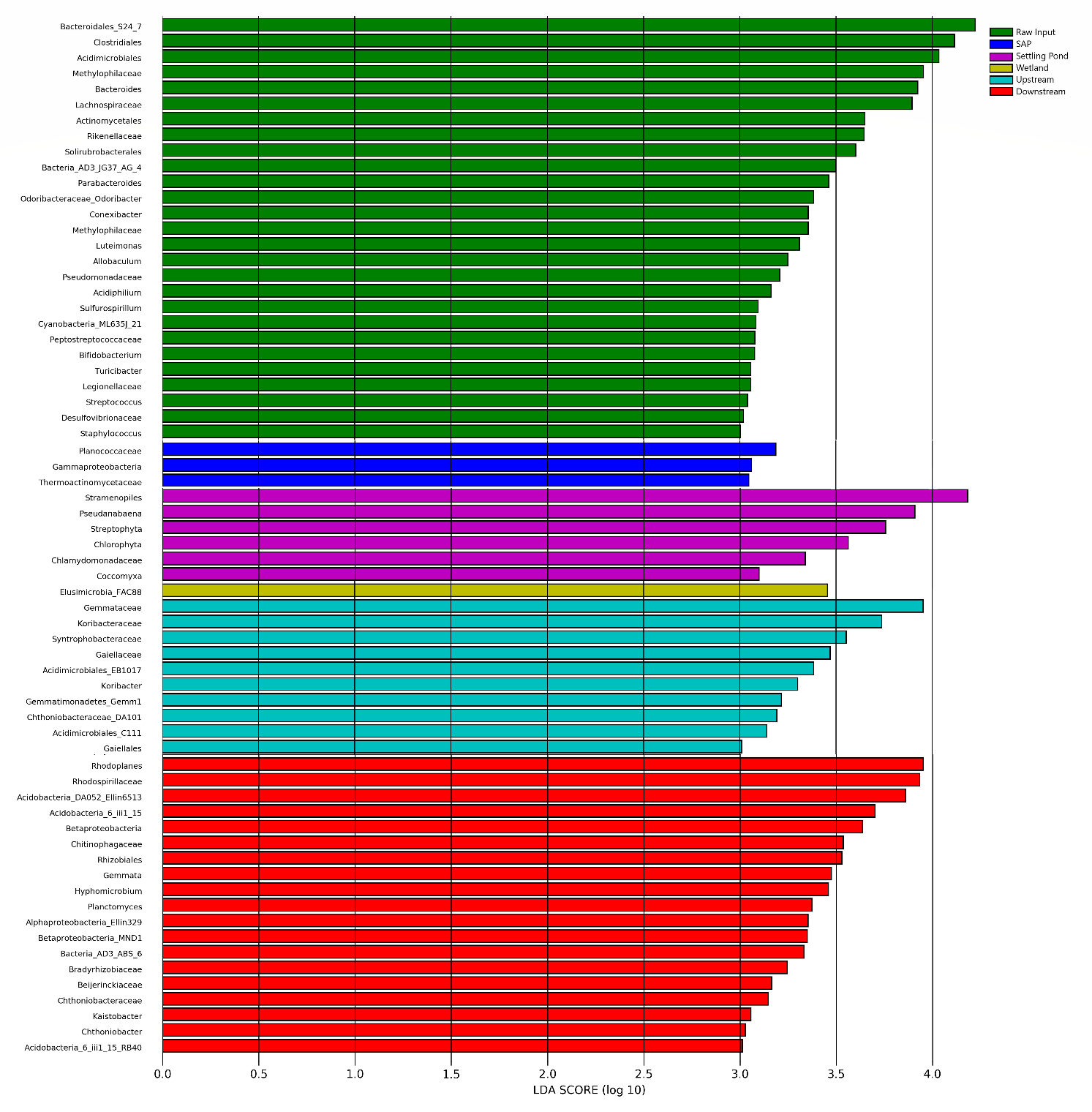


**Figure S5:** LEfSe analysis depicted through a bar graph revealing significantly enriched bacterial taxa (Kruskal-Wallis and Wilcoxon rank sum test alpha=0.05, LDA > 3.0) within each respective sampling site. Length of bars represent the LDA (linear discriminant analysis) score for each taxon. The bar graph exhibits taxa with significant differences in abundance between sites.


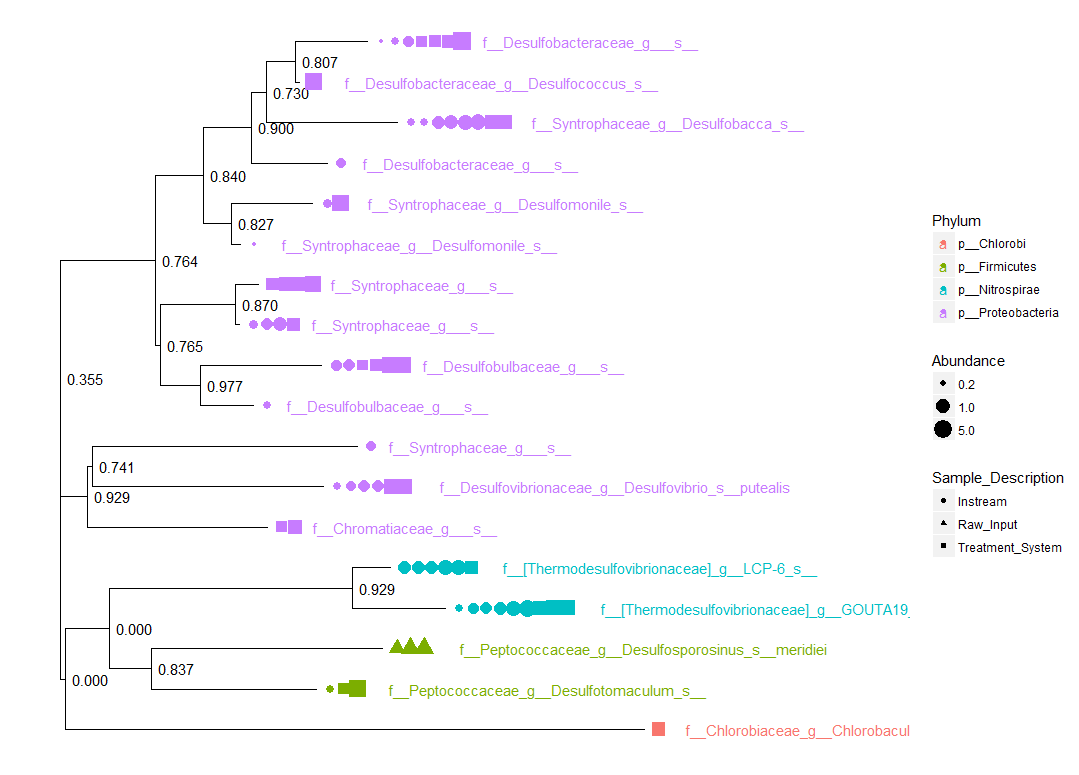


**Figure S6:** Dendrogram of sulfate reducing and sulfur oxidizing bacteria identified throughout Middle Branch remediation system and its receiving stream was generated using the Phyloseq package in R. Node labels represent the highest available resolution of identified bacteria. The colored node labels are representative of the phylum as indicated by the figure legend. Relative abundance was visualized through the different sized shapes, where smaller shapes indicate lower relative abundance and larger shapes suggest higher relative abundance. The shapes are a visual representation of which sites these bacteria were identified within, where circles represent instream, triangles represent raw AMD discharge, and squares represent sites within the remediation system.


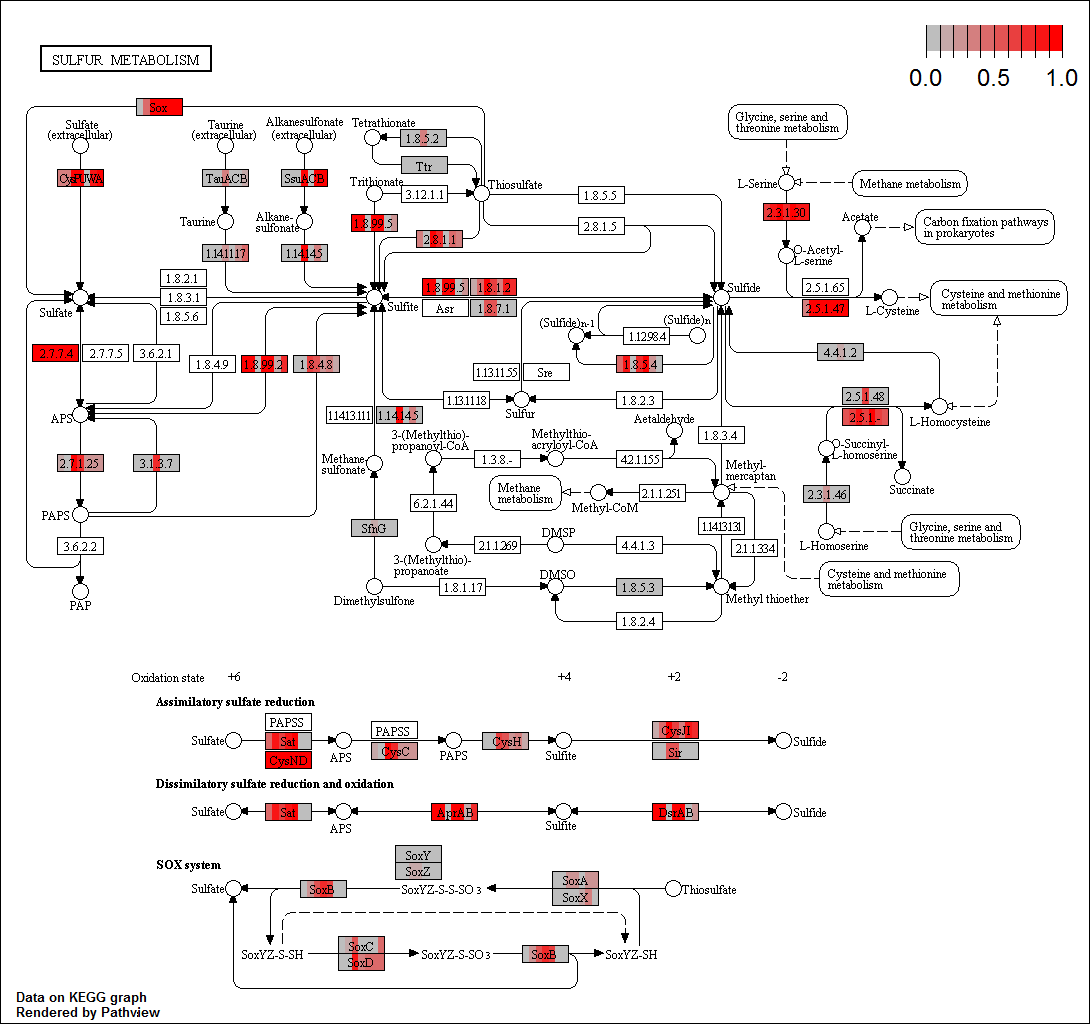


**Figure S7:** KEGG pathway of key genes involved in sulfate reduction and oxidation. Each box represents a gene that is responsible for the transformation of sulfur into various forms. Highlighted subsections within the boxes represent relative abundance of that gene in each site at Middle Branch: SAP1A, SAP1B, settling pond, SAP2B, wetland, upstream, downstream. Grey colors signify little to no abundance of that gene in a certain site, while red represents high levels of abundance.

**Table S4:** Table comparing bacterial richness using alpha diversity metrics. Alpha diversity was calculated for both SAPS sites. Alpha diversity measures didn’t reveal significant differences in species richness between sediment and water samples from SAPS (P > 0.05). The table contains mean and standard deviation values for each alpha diversity metric.

| **Sample Type** | **Alpha Diversity Metrics** | | | |
| --- | --- | --- | --- | --- |
|  | Observed | Chao1 | heipE | PTDTree |
| Water  N = 3 | 1211 ± 474 | 2330 ± 762 | 0.219 ± 0.135 | 102 ± 31.3 |
| Sediment N = 3 | 1700 ± 241 | 2810± 381 | 0.312 ± 0.056 | 149 ± 19.8 |


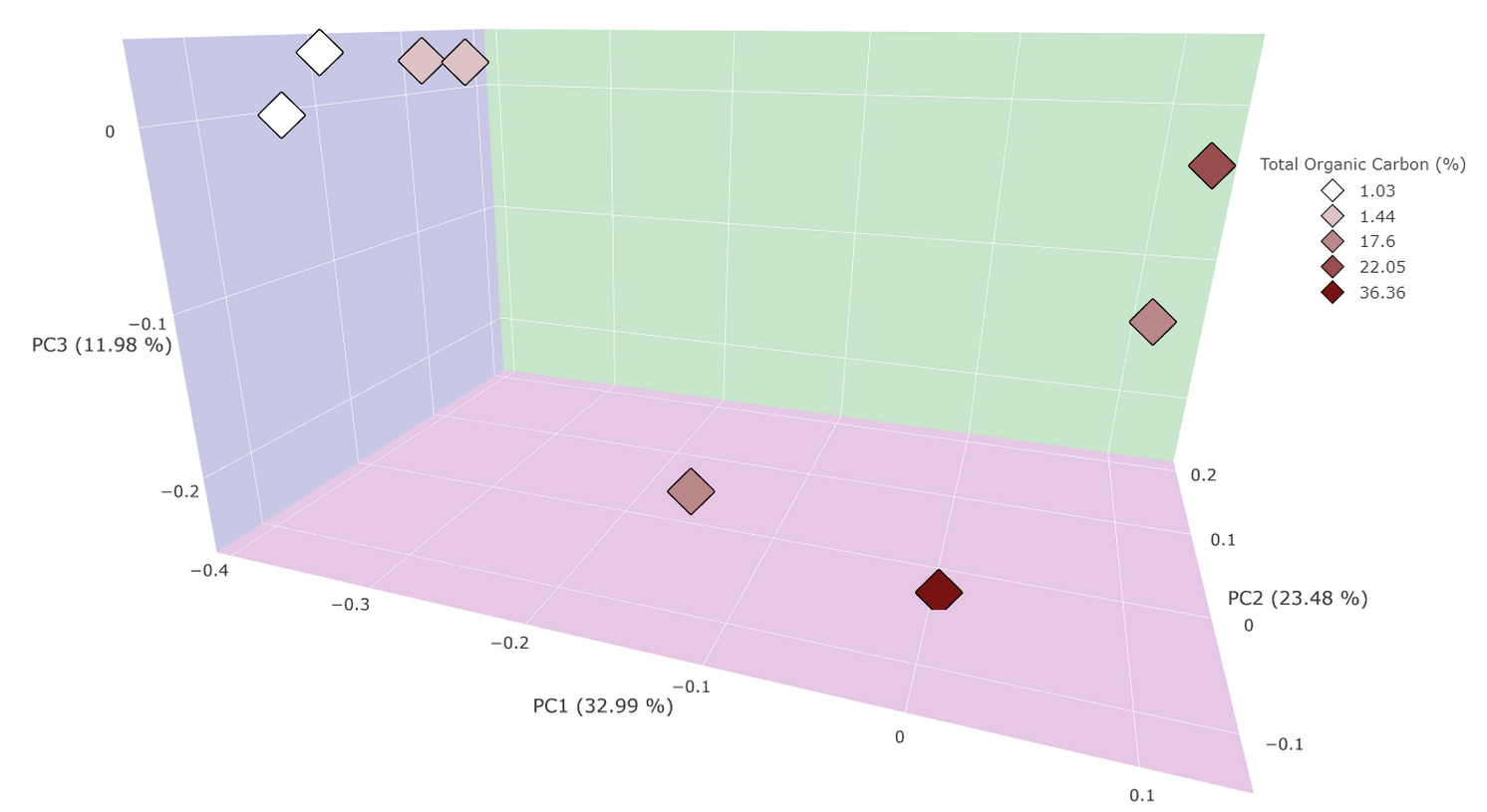


**Figure S8:** Weighted principal coordinates axes (PCoA) plots were generated to visualize community differences between sediment samples of increasing carbon composition.  Total organic carbon % was found to cause the greatest amount of variation in microbial community structure between sediment samples (55.2%, Adonis P <.001). Samples collected within the treatment system (right cluster) appear to have greater carbon concentrations in comparison to samples collected upstream and downstream (left cluster) of the remediation system.
